# Association of vitamin D_3_ with alveolar bone regeneration in dogs

**DOI:** 10.1111/jcmm.12460

**Published:** 2015-03-08

**Authors:** Hsiang-Hsi Hong, Tzung-Hai Yen, Adrienne Hong, Ting-An Chou

**Affiliations:** a Department of Periodontics, Chang Gung Memorial Hospital and Chang Gung UniversityLinkou, Taiwan; b School of Dental Technology, College of Oral Medicine, Taipei Medical UniversityTaipei, Taiwan; c Department of Nephrology and Division of Clinical Toxicology, Chang Gung Memorial Hospital and Chang Gung UniversityLinkou, Taiwan; d Department of Integrative Biology, University of CaliforniaBerkeley, CA, USA

**Keywords:** alloplast, bone regeneration, calcitriol, cholecalciferol, vitamin D_3_, ridge preservation

## Abstract

Designed sockets prepared on the mandibles of nine Beagle dogs were divided into three groups: Calcitriol +Alloplast, Alloplast and Empty. Five of the nine dogs received Vit.D_3_ and calcium supplement (Vit.D/Ca group), while the other four dogs without supplements were assigned to Non-Vit.D/Ca group. After 4 weeks, the extent of vertical ridge resorption (VRR), bone density (density), new bone formation (NBF) and implant stability quotient (ISQ) were measured. Following systemic Vit.D/Ca administration, the Empty subgroup showed significant differences from the Calcitriol + Alloplast subgroup on variants NBF/Density/VRR and the Alloplast subgroup on items NBF/Density/ISQ/VRR. Alternatively, the Calcitriol + Alloplast subgroup revealed higher values of NBF/Density/ISQ (*P* < 0.001) and a lower VRR value (*P* = 0.001) than the Alloplast subgroup. Although there were no significant differences in NBF (*P* = 0.349), density (*P* = 0.796), ISQ (*P* = 0.577) and VRR (0.979) comparisons on alloplast treatment between the Vit.D/Ca and Non-Vit.D/Ca groups, local application with Calcitriol + Alloplast demonstrated better NBF/Density/ISQ (*P* = 0.02 to <0.001) effects than which of Alloplast subgroups. Consequently, the results showed that both systemic and local vitamin D_3_ treatment might accelerate bone regeneration in dogs. Within the using dose, systemic vitamin D_3_ treatment displayed a superior stimulating effect than local vitamin D_3_ application did.

## Introduction

Many human and animal trials have studied the effects of ridge preservation by placing various bone grafts and substitutes into extraction sockets or created defects. However, the bone quantity and quality at the regenerated sites have been inconsistent to date.

Local biphasic calcium phosphate ceramic (BCP) application into the fresh extraction sockets of dog promoted the morphologic preservation of the alveolar ridge significantly in 3 months 1. Synthetic bone grafts were referred to serve as scaffolds for bone formation, maintain space for bone growth, and promote wound healing by stabilizing blood clots 2. However, the osseoinduction potential by using synthetic bone grafts alone is limited 3. Several studies have documented that growth factors can enhance bone regeneration 3–5 and the regenerative capability of growth factors depends greatly on the method of being applied 6. More than enhanced the bone regeneration significantly, a combination of growth factors and synthetic bone ceramic (or polymeric) grafting materials allowed the sustained release and reduced the dosage of growth factors.

An early report declared irrelevancy between vitamin D metabolites and bone formation in rats 7. However, many animal models supported the positive correlation between bone healing and systemic vitamin D_3_ supplement, in which the vitamin D_3_ metabolites promoted the repair of bone fractured and increased the callus strength in chicks, rats and rabbits 8–11. Human studies demonstrated that vitamin D_3_ and calcium supplementation not only reduced hip bone resorption and fracture rates 12–15 but they also increased vertebral bone density and total body calcium in post-menopausal women with vitamin D_3_ supplementation of 10 μg/day and an adjusted calcium intake of 1000 mg/day 16. However, the studies that explore the distinct effect of systemic vitamin D_3_ supplement and local vitamin D_3_ application on bone healing after traumatic fracture, pathologic defects, or surgically created defects in large animal are rare.

A dog's study reported that the combination of calcium supplementation and vitamin D_3_ might have systemic effects in accelerating alveolar bone regeneration 17; however, the local effect of vitamin D_3_ relevance remains undetermined. Recent evidence suggested that calcitriol and its associated metabolites might stimulate bone mineralization directly: *In vitro* studies have revealed that the vitamin D receptor is present in osteoblasts and that calcitriol directly affects the ability of osteoblasts to regulate the expression of several genes. Subsequently, the proliferation of osteoblasts and the production of type I collagen, alkaline phosphatase and osteocalcin intensify; these verifications provide further support for the regulatory function of the vitamin D_3_ in bone formation and mineralization 16,18,19. In addition, incorporating a biodegradable polyurethane bone graft substitutes with vitamin D_3_ could enhance bone regeneration of bicortical defects in the iliac crest of oestrogen-deficient sheep 20. On the basis of these studies, it has been suggested that calcitriol might exert a local effect on alveolar bone regeneration as well. The first aim of the current experiment was to explore the regenerative possibility of active vitamin D_3_ (calcitriol), which was applied locally to surgically created defects during the experimental period. The other aim of this trial was to compare the possible regeneration effects of systemically vitamin D_3_ administration (containing calcium and cholecalciferol supplement) to that of localized vitamin D_3_ (calcitriol) application on the created defects in alveolar bone of dogs during the early healing stage.

## Materials and methods

### Animals

The animal ethical committee of Chang Gung Memorial Hospital (CGMH) granted this research protocol. Nine Beagle dogs that were approximately 1 year old and weighed 10–15 kg each were employed in the study.

### Agents and biomaterials

Bonagraft (San Chung, New Taipei City, BioTech One, Taiwan) a synthetic BCP alloplast (HA/β-TCP) containing 60% hydroxyapatite (HA)/40% beta tri-calcium phosphate (β-TCP) of 250–500 μm in size, was adopted to preserve the prepared ridges in this study. Calcijex® (Hospira Inc. for Abbott Laboratories, Rocky Mount, NC, USA) is an active form of vitamin D_3_ (calcitriol; 1α, 25(OH)_2_D_3_), which contains 1 ml of 80 IU calcitriol. One millilitre of Calcijex® mixed with 0.5 g of alloplast was inserted into a defect. In addition, Calcijex® was further injected submucously around the Calcitriol + Alloplast sites every 7 days on the right mandibles of Vit.D/Ca and Non-Vit.D/Ca groups. In the Vit.D/Ca group, every dog was fed additionally six oral Tablets of Bio-cal® per day that each Tablet was composed of 802 mg tri-calcium phosphate (TCP, equivalent to 300 mg calcium) and 1.56 μg cholecalciferol. Both Bio-cal® and Calcijex® were provided by the CGMH pharmacy.

### Experimental design

The dogs were randomly divided into two groups. Five dogs receiving Bio-cal® supplementation were assigned to the treatment group (Vit.D/Ca group); the other five dogs without Bio-cal® supplement were assigned to the control group (Non-Vit.D/Ca group), however, one of the five dogs was excluded because of inadequate bodyweight. The sockets on each mandible were divided into three treatment modalities: four sockets on the right side were grafted with 1 ml of Calcijex® and 0.5 g of HA/β-TCP (Calcitriol + Alloplast subgroup); two of the four sockets on the left side were randomly grafted with HA/β-TCP (Alloplast subgroup) and the other two defects were left empty (Empty subgroup).

### Surgical procedure

**Figure 1:**
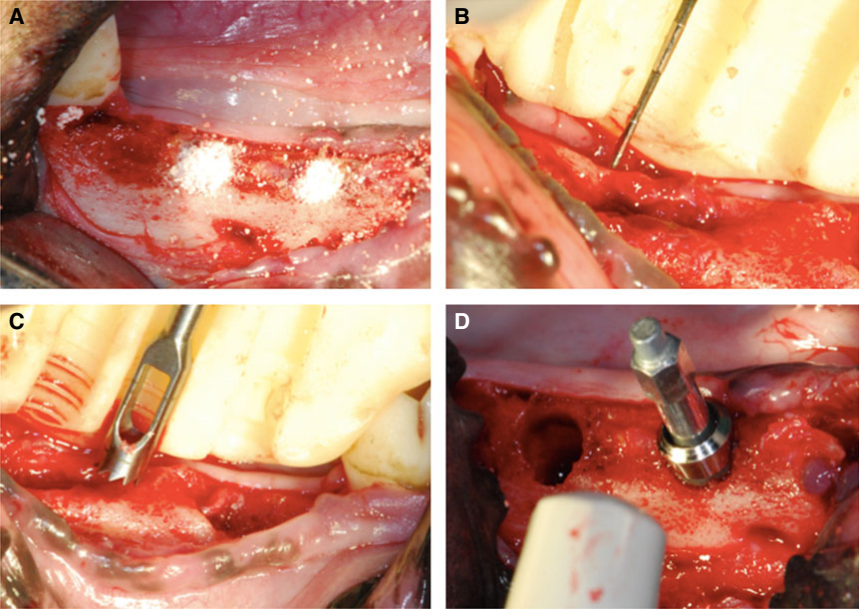
Cylindrical-defect osteotomy and 4 weeks re-entry surgeries were applied for clinical and histological analysis. (A) Standardized sockets were prepared in both sides of the mandible. The socket defects were divided into three local treatment modalities. (B) The amount of vertical ridge resorption was evaluated according to a surgical stent. (C) The 3-mm-diameter and 6-mm-deep bone cores were drawn with a 4-mm-diameter trephine bur from the sites determined by a surgical stent. (D) A 4.8-mm-diameter with 6-mm-length implant was inserted and the ISQ was detected.

All surgical procedures were performed in the animal laboratory of CGMH. To reduce salivation, a subcutaneous premedication of atropine (0.05 mg/kg s.c.) was applied to the dogs prior to surgery. Fifteen minutes later, the generalized anaesthesia were inducted intramuscularly with Zoletil 50® (10–15 mg/kg) and maintained on 0.5–2% isofluorane/oxygen *via* a cuffed endotracheal tube. Veterinarians in the animal laboratory also performed the pre-operative analgesia procedure. Under aseptic routines, mandibular nerve block (2% lidocaine hydrogen chloride at a total dose of 2 mg/kg) was administered and the premolars on both sides of the mandible were extracted during the initial stage of the study. Three months later, four standardized cylindrical sockets with 4.0 mm in diameter and 6 mm in depth were prepared on both sides of the mandible after flap operation. The socket defects were divided into three local treatment modalities, as described above (Fig.1A).

### Data collection

After a 4-week healing period, the amount of vertical ridge resorption (VRR) was evaluated according to a surgical stent (Fig.1B). The bone cores 3.0 mm in diameter and 6 mm deep were drawn with a 4.0 mm outer diameter trephine bur from the sites determined by the surgical stent, and placed in a fixative for histomorphometric analysis (Fig.1C). Subsequently, an implant (ITI Straumann®) with 4.8 mm in diameter and 6.0 mm in length was inserted and the implant stability quotient (ISQ) was measured with Osstell® (Osstell AB, Gamlestadsvägen 3B, SE 415 02 Göteborg, Sweden) immediately after implant placement by performing resonance frequency analysis (Fig.1D). To reduce experimental error, the same investigators repeated all measurements and averaged the two closest values.

### Post-surgical procedure

After surgery, the dogs were placed in cages warmed on a circulating water heating pad and kept under observation for signs of distress until they were able to move normally. Post-operative analgesia with an oral non-steroidal anti-inflammatory was administered every 8–12 hrs for 2 days. A soft-pellet diet was fed to these dogs throughout the survey.

The dogs assigned to the Vit.D/Ca group were given six Tablets of Bio-cal® every day for 4 weeks 21. At the end of the experiment, the dogs were killed with an overdose of Pentothal Natrium perfused through the carotid arteries, with a fixative containing a mixture of 5% glutaraldehyde and 4% formaldehyde.

### Cone beam computed tomography analysis

Cone beam computed tomography (CBCT) scans with 0.25-mm-thick slices were obtained for each bone block point on an iCAT CBCT machine (Imaging Sciences International, Hatfield, PA, USA). An installed computed tomography program determined the bone density, which was described by a Hounsfield unit (HU) value.

### Histotechnical preparation and histometric analysis

Each block, 3.0 mm in diameter and 6 mm tall, was decalcified in ethylenediaminetetraacetic acid, dehydrated in increasing concentrations of ethanol, embedded in paraffin, and cut horizontally by the pathology department. The sections were stained in haematoxylin and eosin. The middle sections of the bony cores, representing the central portion (in a subcrestal cross-section 3 mm in area), from each group were selected and subjected to histological evaluation. Histological images were taken using a digital camera (Nikon Diaphot 300, Southern Micro Instruments, Atlanta, GA, USA) attached to an inverted microscope (Olympus BX51 Microscope, Hatagaya, Shibuya-ku, Tokyo, Japan) equipped with a ×10 objective lens. The area of new bone formation (NBF) was measured in pixels using digital imaging software (Image J, National Institutes of Health, Bethesda, MD, USA) and expressed as a percentage of NBF (Fig.1).

### Statistical analysis

Group means and standard deviations were calculated for each measured parameter. Information regarding the percentage of NBF, bone density (Hu) at preparation sites, ISQ and VRR achieved from surgical sites was evaluated using anova and LSD tests. The anova and LSD tests approach were employed to determine whether a systemic supplement of Vit.D/Ca and the three various local treatments affected bone defect regeneration. A *P* < 0.05 was considered significant. As anova and LSD tests account for possible correlations of bone regeneration at different locations within a patient, they can also be used to measure the significance of possible covariates. The application of anova and LSD tests in this study were intended to model dependent variables (NBF, density, ISQ and VRR) as a function of local treatment or Vit.D/Ca supplement. Before and after survey, the examined serum items [intact parathyroid hormone (PTH), calcium, phosphorus and 25-OH-D_3_] were monitored by Mann–Whitney test (*P* < 0.05).

## Results

In generalized aspects (Tables1 and 2), the dogs receiving a Vit.D/Ca supplement exhibited more NBF (33.68 ± 14.87% *versus* 22.96 ± 10.30%, *P* = 0.001) and higher bone density (1156.92 ± 438.19 Hu *versus* 890.06 ± 300.76 Hu, *P* = 0.006) than those without Vit.D/Ca supplement. On the other hand, from the viewpoint of local expression, the Empty subgroup achieved higher values of NBF (42.35 ± 12.52%), density (1373.29 ± 367.05 Hu), ISQ (75.06 ± 5.24) and VRR (2.45 ± 0.34 mm) than the other two subgroups (*P* < 0.001). Furthermore, the Calcitriol + Alloplast (HA/β-TCP) subgroup also revealed higher values of NBF (29.92 ± 11.89% *versus* 15.69 ± 5.17%, *P* < 0.001), density (1121.97 ± 287.20 Hu *versus* 584.79 ± 225.05 Hu, *P* < 0.001) and ISQ (73.90 ± 5.18 *versus* 65.29 ± 7.99, *P* < 0.001) than that of the Alloplast subgroup.

**Table 1:** Results of taking systemic vitamin D_3_ or not on the tested variables of the alveolar bone regeneration in dogs

Variables	Vit.D/Ca			Non-Vit.D/Ca		
	Calcitriol + Alloplast	Alloplast	Empty	Calcitriol + Alloplast	Alloplast	Empty
NBF (%)	35.24 ± 11.21	17.49 ± 5.04	48.19 ± 12.36	23.28 ± 9.28	13.13 ± 4.50	33.58 ± 6.40
Density (Hu)	1273.68 ± 236.56	598.28 ± 233.04	1518.18 ± 383.44	932.34 ± 229.20	565.53 ± 229.91	1155.96 ± 218.00
ISQ (0–100)	73.58 ± 5.51	64.60 ± 8.91	76.00 ± 4.80	74.31 ± 4.88	66.29 ± 7.02	73.67 ± 6.02
VRR (mm)	1.17 ± 0.24	1.46 ± 0.17	2.27 ± 0.27	1.42 ± 0.19	1.46 ± 0.13	2.72 ± 0.23

NBF, new bone formation; ISQ, implant stability quotient; VRR, vertical ridge resorption.

**Table 2:** Generalized aspects and local expressions on the examined variants of the alveolar bone regeneration in dogs

	NBF (%)	Density (Hu)	ISQ	VRR (mm)
Generalized aspects (Pooling the data of Calcitriol/alloplast, Alloplast, Empty groups)				
Vit.D/Ca	33.68 ± 14.87	1156.92 ± 438.19	71.83 ± 7.64	1.50 ± 0.50
Non-Vit.D/Ca	22.96 ± 10.30	890.06 ± 300.76	72.24 ± 6.45	1.70 ± 0.56
*P*-value	0.001**	0.006**	0.817	0.127
Local expressions (Pooling the data of Vit.D/Ca and Non-Vit.D/Ca groups)				
Calcitriol + Alloplast	29.92 ± 11.89	1121.97 ± 287.20	73.90 ± 5.18	1.28 ± 0.25
Alloplast	15.69 ± 5.17	584.79 ± 225.05	65.29 ± 7.99	1.45 ± 0.15
Empty	42.35 ± 12.52	1373.29 ± 367.05	75.06 ± 5.24	2.45 ± 0.34
*P*-value	<0.001***	<0.001***	<0.001***	<0.001***

Significance differences among various subgroups were examined using trend estimation. Following systemic administration, the Vit.D/Ca group showed higher value of NBF (*P* = 0.001) and density (*P* = 0.006) than Non-Vit.D/Ca group.

ISQ, implant stability quotient; NBF, new bone formation; VRR, vertical ridge resorption.

** *P* < 0.01

*** *P* < 0.001.

**Figure 2:**
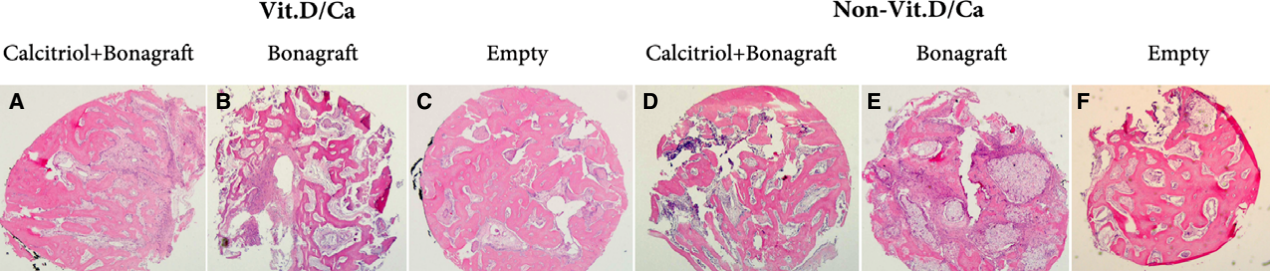
The effects of vitamin D_3_ on alveolar bone regeneration in dogs were examined under microscopic observation (haematoxylin and eosin stain, original magnification ×10). (A) Empty defect in an individual receiving Vit.D/Ca supplement. (B) Empty defect in an individual without Vit.D/Ca supplement. (C) Calcitriol + alloplast defect in an individual receiving Vit.D/Ca supplement. (D) Calcitriol + alloplast defect in an individual without Vit.D/Ca supplement. (E) Alloplast defect in an individual receiving Vit.D/Ca supplement. (F) Alloplast defect in an individual without Vit.D/Ca supplement.

As shown in Figure2 and Table3, non-significant NBF difference was noted in the Alloplast subgroups between Vit.D/Ca and Non-Vit.D/Ca groups (*P* = 0.349), neither were the Empty subgroup of Non-Vit.D/Ca group and the Calcitriol/Alloplast subgroup of Vit.D/Ca group (*P* = 0.706). Comparing to the Alloplast subgroup of Vit.D/Ca group, the Calcitriol/Alloplast subgroup of Non-Vit.D/Ca group demonstrated a statistic effect of non-difference (*P* = 0.131). Except that, all subgroups demonstrated significant NBF differences to each other, such as local treatment with Calcitriol + Alloplast caused significantly greater NBF value than that occurred in the Alloplast variants no matter with Vit.D/Ca (*P* < 0.001) or without Vit.D/Ca (*P* = 0.020) supplement.

**Table 3:** Comparison of the new bone formation (NBF) among various subgroups

NBF	Vit.D/Ca			Non-Vit.D/Ca		
	Calcitriol + Alloplast	Alloplast	Empty	Calcitriol + Alloplast	Alloplast	Empty
(%)	35.24 ± 11.21	17.49 ± 5.04	48.19 ± 12.36	23.28 ± 9.28	13.13 ± 4.50	33.58 ± 6.40
Vit.D/Ca						
Calcitriol + Alloplast		<0.001***	0.001**	<0.001***	<0.001***	0.706
Alloplast			<0.001***	0.131	0.349	0.001**
Empty				<0.001***	<0.001***	0.004**
Non-Vit.D/Ca						
Calcitriol + Alloplast					0.020*	0.025*
Alloplast						<0.001***
Empty						

Significant differences among most various groups were examined using anova and LSD tests.

NBF, new bone formation.

*** *P* < 0.001

** *P* < 0.01

* *P* < 0.05.

The relationship of the density among various subgroups showed that non-significant difference was found in the Alloplast subgroups between Vit.D/Ca and Non-Vit.D/Ca groups (*P* = 0.796), neither was the Empty subgroup of Non-Vit.D/Ca group and the Calcitriol + Alloplast subgroup of Vit.D/Ca group (*P* = 0.327). Furthermore, in Non-Vit.D/Ca group, the Empty subgroup and the Calcitriol/Alloplast subgroup did not display a statistic difference (*P* = 0.073). However, both Calcitriol + Alloplast and Empty subgroups revealed higher Hu values than which of Alloplast subgroups in both Vit.D/Ca group and Non-Vit.D/Ca group (from *P* < 0.001 to *P* = 0.002). The Empty subgroups presented a significant density difference between Vit.D/Ca group and Non-Vit.D/Ca group (*P* = 0.009). In Vit.D/Ca group, the Empty subgroup also displayed a statistically higher density than that of the Calcitriol/Alloplast subgroup (*P* = 0.021, Table4).

**Table 4:** Relationship of the Density among various subgroups

Density	Vit.D/Ca			Non-Vit.D/Ca		
	Calcitriol + Alloplast	Alloplast	Empty	Calcitriol + Alloplast	Alloplast	Empty
(Hu)	1273.68 ± 236.56	598.28 ± 233.04	1518.18 ± 383.44	932.34 ± 229.20	565.53 ± 229.91	1155.96 ± 218.00
Vit.D/Ca						
Calcitriol + Alloplast		<0.001***	0.021*	<0.001***	<0.001***	0.327
Alloplast			<0.001***	0.002**	0.796	<0.001***
Empty				0.001**	<0.001***	0.009**
Non-Vit.D/Ca						
Calcitriol + Alloplast					0.002**	0.073
Alloplast						<0.001***
Empty						

* *P* < 0.05

** *P* < 0.01

*** *P* < 0.001 by anova and LSD tests.

Comparing to the Empty subgroups, adding Alloplast diminished the ISQ in Vit.D/Ca group and Non-Vit.D/Ca group significantly (*P* < 0.001 and *P* = 0.034 respectively, Table5). In addition, systemic cholecalciferol/Ca supplement did not up-regulate more ISQ values for all three subgroups than Non-Vit.D/Ca group (*P* = 0.720 in Calcitriol + Alloplast, *P* = 0.577 in Alloplast and *P* = 0.471 in Empty subgroups). However, local calcitriol applications compensated the compromised ISQ that originated from alloplast appendage (*P* < 0.001 in Vit.D/Ca group and *P* = 0.005 in Non-Vit.D/Ca group respectively) and derived to the ISQs comparable to Empty groups (*P* = 0.326 in Vit.D/Ca group and *P* = 0.826 in Non-Vit.D/Ca group).

**Table 5:** Evaluation of the implant stability quotient (ISQ) among various subgroups

ISQ	With Vit.D/Ca			Non-Vit.D/Ca		
	Calcitriol + Alloplast	Alloplast	Empty	Calcitriol + Alloplast	Alloplast	Empty
(0–100)	73.58 ± 5.51	64.60 ± 8.91	76.00 ± 4.80	74.31 ± 4.88	66.29 ± 7.02	73.67 ± 6.02
Vit.D/Ca						
Calcitriol + Alloplast		<0.001***	0.326	0.720	0.008**	0.974
Alloplast			<0.001***	<0.001***	0.577	0.006**
Empty				0.509	0.002**	0.471
Non-Vit.D/Ca						
Calcitriol + Alloplast					0.005**	0.826
Alloplast						0.034*
Empty						

* *P* < 0.05

** *P* < 0.01

*** *P* < 0.001 by anova and LSD tests.

Ridge preservation by alloplast, no matter with or without calcitriol application, resulted in less VRR than Empty subgroups (*P* < 0.001) in both Vit.D/Ca and in Non-Vit.D/Ca groups. Systemic Vit.D/Ca supplement could lessen VRR in Calcitriol + Alloplast (*P* < 0.001) and Empty (*P* < 0.001) subgroups than which of Non-Vit.D/Ca group. However, local calcitriol application only affected the VRR effect of alloplast in Vit.D/Ca group (*P* < 0.001), but not in Non-Vit.D/Ca group (*P* = 0.695, Table6).

**Table 6:** Variation in the vertical ridge resorption (VRR) among various subgroups

VRR	With Vit.D/Ca			Non-Vit.D/Ca		
	Calcitriol + Alloplast	Alloplast	Empty	Calcitriol + Alloplast	Alloplast	Empty
(mm)	1.17 ± 0.24	1.46 ± 0.17	2.27 ± 0.27	1.42 ± 0.19	1.46 ± 0.13	2.72 ± 0.23
Vit.D/Ca						
Calcitriol + Alloplast		0.001**	<0.001***	0.001**	0.004**	<0.001***
Alloplast			<0.001***	0.636	0.979	<0.001***
Empty				<0.001***	<0.001***	<0.001***
Non-Vit.D/Ca						
Calcitriol + Alloplast					0.695	<0.001***
Alloplast						<0.001***
Empty						

** *P* < 0.01

*** *P* < 0.001 by anova and LSD tests.

Comparing to the baseline examination, the prescribed Vit.D/Ca supplement decreased serum levels of intact PTH (*P* = 0.018), 25-OH-D_3_ (*P* = 0.003) and phosphorus (*P* = 0.042) significantly. On the other hand, only serum 25-OH-D_3_ levels were influenced significantly by local calcitriol application (*P* = 0.005).

In contrast to systemic Vit.D/Ca supplement, local calcitriol application revealed a significant increase in serum intact PTH levels (*P* = 0.019) and a significant decrease in serum 25-OH-D_3_ levels (*P* = 0.027, Table7).

**Table 7:** Differentiation of the blood tests in dogs taking systemic Vit.D/Ca supplement to those receiving local Vit.D_3_ (Non-Vit.D/Ca) application

Dog number	Before study (n = 9)	Sys Vit.D/Ca + Loc Vit.D_3_ (n = 5)	Loc Vit.D_3_ (n = 4)	*P*-value
Tested items (normal range)				
	11.01 ± 0.105		11.02 ± 0.538	0.937
	11.01 ± 0.105	11.28 ± 0.421		0.222
		11.28 ± 0.421	11.02 ± 0.538	0.539
Intact PTH (1–4 pg/ml)	1.92 ± 1.109		2.53 ± 0.350	0.118
	1.92 ± 1.109	0.44 ± 0.984		0.018*
		0.44 ± 0.984	2.53 ± 0.350	0.019*
Serum Phos (2.1–6.3 mg/dl)	5.43 ± 0.482		4.75 ± 0.695	0.139
	5.43 ± 0.482	4.7 ± 0.644		0.042*
		4.7 ± 0.644	4.75 ± 0.695	0.902
25-OH-D_3_ (25–200 nmol/l)	31.67 ± 0.84		27.45 ± 0.412	0.005*
	31.67 ± 0.84	28.68 ± 0.672		0.003*
		28.68 ± 0.672	27.45 ± 0.412	0.027*

Sys Vit.D/Ca + Loc Vit.D_3_, Systemic Vit.D/Ca + Local Vit.D_3_ treatment; Loc Vit.D_3_, Local Vit.D_3_ application; Intact PTH, Intact parathyroid hormone; Serum Ca, Serum Calcium level; 25-OH-D_3_, Serum 25-OH-vitamin D_3_; Serum Phos, Serum Phosphorus level.

* *P* < 0.05 by Mann–Whitney test.

## Discussion

Up to now, limited evidence are available to support the idea of taking vitamin D_3_ and calcium supplementation (Vit.D_3_/Ca) may stimulate early bone healing and accelerate bone quality and quantity in the alveolar sockets of dogs. The results of this study correspond to some previous reports and demonstrate that taking Vit.D/Ca supplementation during the early stage of socket healing promotes more NBF, higher bone density and less VRR when compared to a non-supplement group (Table2). However, taking Vit.D/Ca supplement does not contribute to enhancing the implant stability in the investigational areas of this study. The fact may be explained by that the newly formed bone is not mature enough to response the ISQ examination. A longer healing period might be required to promote a better implant stability and achieve a higher ISQ value.

Although the outcome of compromised ISQ value, less NBF and lower bone density that associated with the grafting HA/β-TCP alloplast are found on both Vit.D/Ca and Non-Vit.D/Ca groups; local calcitriol application demonstrates an improving effect on NBF (*P* = 0.020, Table3), bone density (*P* = 0.002, Table4) and ISQ value (*P* = 0.005, Table5) than which of the HA/β-TCP grafted sockets. The benefit of local calcitriol application on bone regeneration in this observation may be rationalized partially by previous *in vitro* studies 16,18,19.

When exploring the effect of local vitamin D_3_ delivery on the supporting bone around the teeth after orthodontic treatment, the findings of studies concluded that the use of calcitriol might eventually enhance bone remodelling or regeneration of the supporting bone 22–25. Meanwhile, a chicken study noted that certain vitamin D derivatives might possess various healing properties and concluded that 24,25-dihydroxyvitamin D_3_ might increase bone healing and prevent the complication rate of non-union. Although the role of bone-wax was not explored, calcitriol decreased callus strength when 50 mg of bone-wax containing 5 μg of calcitriol or 24,25-dihydroxyvitamin D_3_ was applied on the fractured site 26. Therefore, the local effect of vitamin D_3_ on bone regeneration remains controversial. Disparate methods and materials may account for the variation; the present study does not find the corresponding clinical or microscopic side effects after local calcitriol application to concur the previous evidence 27. According to our knowledge, although local calcitriol effect without carrier on empty sockets was not tested, this study is the first report to suggest that local application of active vitamin D_3_ acts as a stimulator of early bone healing in the alveolar sockets of dogs.

Various bone grafts and substitutes have been utilized effectively to preserve the post-extraction sockets 28,29, such as demineralized frozen-dried bone allograft 28,30,31, mineralized frozen-dried bone allograft 32, deproteinized bovine bone 33, alloplastic polymers 34 and bioactive glasses 31. The Bio-Oss Collagen augmented defect exhibited less wound shrinkage than the non-augmented defect (0.1 mm *versus* 0.8 mm of hard-tissue bridge) 35. A previous canine study observed that ridge resorption over 8 weeks following extraction resulted in 1.39 mm of bone loss in an extraction socket filled with calcium sulphite and platelet-rich plasma, and 1.32 mm of bone loss in a socket filled with calcium sulphite, and 2.77 mm of bone loss in a socket without any material filling 36. Barone's group observed lingual vertical bone loss of 0.4 mm in a socket filled with porcine cortico-cancellous bone and collagen membrane, and 3 mm of bone loss in a socket without material filling 37. However, not only were the post-extraction alveolar ridge dimensions partially preserved by some of these bone substitutes but also the quantity and quality of bone tissue formed in the socket were also variable. This may have been the result of various wound sizes and variable healing time intervals. In the present investigation, the alloplast applied delayed NBF, reduced bone density, and decreased ISQ reading during the early stage of socket healing. These results are comparable to previous animal studies 38–40, which resulted in reduced NBF and delayed initial healing in fresh extraction sockets filled with deproteinized bovine bone in combination with collagen. The assumption is that the employed bone substitute provided a scaffold for space maintenance and osteoconduction at the expense of compromising blood supply and interfering with the normal healing process in the tested areas. Hence, bone regeneration with a bone substitute may help to retain the ridge contour better than spontaneous healing, but may also require a longer healing period to generate a better quality and quantity of bone, which has an impact on subsequent implant surgery as dental implants rely on the availability of vital bone to achieve osseointegration 41. Similarly to the previous studies 28,30,31,42, current investigation supports that grafted alloplast minimize vertical resorption of the alveolar ridge but prevent the normal healing process of the extraction socket during the early-phase of bone healing.

The results demonstrate that systemic Vit.D/Ca supplement seems does not assist to increase more NBF (*P* = 0.349) and density (*P* = 0.796) in Alloplast subgroup than without taking supplement. On the other hand, employing Vit.D/Ca supplement and/or local calcitriol application could up-regulate the diminished NBF (*P* < 0.001 in Vit.D/Ca group and *P* = 0.020 in Non-Vit.D/Ca group respectively, Table3) and density (*P* < 0.001 in Vit.D/Ca group and *P* = 0.002 in Non-Vit.D/Ca group respectively, Table4) caused by HA/β-TCP alloplast graft. Systemic Vit.D/Ca supplement also enhance NBF and density effects in Calcitriol + Alloplast (*P* < 0.001 in Tables3 and 4) and Empty (*P* = 0.004 in Table3 and 0.009 in Table4) subgroups (Tables3 and 4).

Consist with other studies, the results of this study support the theory that alloplast may impede NBF, healing bone density and implant stability. On the other hand, alloplast assist to reduce the amount of VRR. Our results also confirm that taking systemic Vit.D/Ca supplement may downgrade significantly VRR for Empty (*P* < 0.001) subgroup (Table6). However, systemic Vit.D/Ca supplement seems does not lessen VRR in Alloplast subgroup than without taking supplement (*P* = 0.979). At given dose, local calcitriol application for Alloplast subgroup does not offer a better effect of decreasing VRR than without calcitriol application (*P* = 0.695) in Non-Vit.D/Ca group. Nevertheless, once cholecalciferol/Ca supplement was provided, Calcitriol + Alloplast subgroup exhibit a significant less VRR than Alloplast subgroup (*P* = 0.001) in Vit.D/Ca group.

Generally, the inspected blood variants demonstrate a normally adjustment on both Vit.D/Ca and Non-Vit.D/Ca groups before and after this survey (Table7). However, only serum calcium changes non-significantly following systemic and/or localized vitamin application. The feedback effect of vitamin D_3_ may explain the findings that serum intact PTH, phosphorus and 25-OH-D_3_ decrease significantly subsequent to systemic Vit.D/Ca supplement and local calcitriol taking. However, the inclination of increasing PTH level without calcium supplement on local calcitriol group needed to be decided by further studies. The different characteristics of calcitriol and cholecalciferol/Ca may play the partial roles on the results of local calcitriol group causes a higher intact PTH value than Vit.D/Ca group. We are unsure of the amount of lethal dose of systemic Vit.D/Ca supplement used (1800 mg calcium/day) for a 15 kg dog; the normal range of calcium, vitamin D_3_ and phosphorus blood data imply that the dose of vitamin D_3_ and calcium supplement administered to the dogs was controlled by their metabolism. On the other hand, the intact PTH reading declined significantly to out of reference range indicate that the offered vitamin D_3_ and calcium supplement was an over dose for the tested dogs. Further researches are required to illuminate these findings. Additionally, in comparison to a previous animal study, our results also demonstrate that a local injection of vitamin D_3_ does not cause serum levels of calcium, phosphorus, PTH or 25-OH- D_3_ to shift out of the normal range 27. Though, the applied local calcitriol dose may initiate a significant drop of serum 25-OH- D_3_.

Within the limitations of this animal study, which included observing the early healing characteristics of artificial defects, our results support that local calcitriol application may accelerate NBF, increase bone density and improve implant stability in surgically produced alveolar sockets treated with 40% HA/60% β-TCP alloplast for both non-Vit.D/Ca and Vit.D/Ca groups. Relating the Calcitriol/Alloplast subgroups of Non-Vit.D/Ca and Vit.D/Ca groups, the comparison show that local calcitriol application may produce less NBF, lower bone density and less ridge preservation effects than which systemic vitamin D_3_ can at the given dose. Less amount of accumulated Vitamin D_3_ dose on Non-Vit.D/Ca group may partially explain the finding. The grafted bone substitute preserve the alveolar ridges significantly, however, the undesirable results of less NBF, inferior bone density and compromised implant stability may associate. Both systemic supplements of cholecalciferol/calcium and local calcitriol application seem significantly minimize the influences.

Further studies are required to determine not only a proper amount of systemic vitamin D_3_ and calcium supplement but also the effective dose and method for local vitamin D_3_ delivery. Solid evidence are still necessitated to support the possible effects of vitamin D_3_ on bone regeneration at earlier and later stages in animals and humans.
